# Maternal and Paternal Representations in Assisted Reproductive Technology and Spontaneous Conceiving Parents: A Longitudinal Study

**DOI:** 10.3389/fpsyg.2021.635630

**Published:** 2021-03-17

**Authors:** Marcella Paterlini, Federica Andrei, Erica Neri, Elena Trombini, Sara Santi, Maria Teresa Villani, Lorenzo Aguzzoli, Francesca Agostini

**Affiliations:** ^1^Department of Obstetrics and Pediatrics, AUSL-IRCCS, Reggio Emilia, Italy; ^2^Department of Psychology, University of Bologna, Bologna, Italy; ^3^Department of Obstetrics and Gynecology, Fertility Center, AUSL-IRCCS, Reggio Emilia, Italy

**Keywords:** assisted reproductive technology (ART), mothers, fathers, parental mental representations, infertility, ICSI (intracytoplasmic sperm injection), IVF (*in vitro* fertilization), longitudinal study

## Abstract

Aim of this study was to investigate whether parental mental representations during pregnancy and after delivery differed between parents who conceived after Assisted Reproductive Treatments (ART) and spontaneous conceiving (SC) parents. Effects of specific ART variables (previous ART attempts, treatment type and cause of infertility) were also taken into account. Seventeen ART couples and 25 SC couples were recruited at Santa Maria Nuova Hospital (Reggio Emilia, Italy). At both 32 weeks of gestation (T1) and 3 months postpartum (T2) participants completed the Semantic Differential of the IRMAG, a self-report tool which measures specific domains of mental representations pertaining either individual (Child, Self-as-woman/man, and Partner) or parental (Self-as-parent, Own parent) characteristics. Results showed that ART parents had significantly more positive representations of the child compared to SC parents, while the scores at Partner dimension improved from T1 to T2 for SC parents only. With regards to ART history, scores at the Self-as-woman/man dimension were significantly less positive for ICSI than IVF parents and improved substantially from T1 to T2 only in case of mothers with previous ART attempts and of fathers at the first ART cycle. The representation of own parents increased from T1 to T2 in case of infertility diagnosis due to male factors, while a decrease emerged when infertility was due to female factors. Findings suggest the need to investigate parental mental representations after ART, in order to improve the understanding on the transition to parenthood of infertile couples and to target more specific intervention for parenting support.

## Introduction

Infertility represents a relevant health issue in many countries across the world, so much so that it currently affects about one in eight couples of reproductive age (McLachlan and O’Bryan, 2010). In order to make parenting possible, an increasing number of infertile couples undergoes Assisted Reproductive Treatments (ART), such as *in vitro* fertilization (IVF) or intracytoplasmic sperm injection (ICSI; Mascarenhas et al., 2012; Ferraretti et al., 2017).

Nevertheless, the recourse to ART leads to potential psychological consequences particularly for women, who may feel a sense of loss, anxiety, depression, and frustration throughout different treatment phases (Fassino et al., 2002; Hammarberg et al., 2008; Monti et al., 2009, 2015; Vitale et al., 2017; Agostini et al., 2018; Allan et al., 2019). Negative consequences could also occur with regards to men’s psychological well-being, with an increased risk of emotional problems such as elevated levels of anxiety and indirect aggression (Hjelmstedt and Collins, 2008; Pinto et al., 2017). Despite the psychological impact seems to be greater for women, as they show higher levels of anxious and depressive symptoms than men (El Kissi et al., 2013; Monti et al., 2015), the investigation of both maternal and paternal psychological state should always be included in the assessment of couples undergoing ART.

The psychological impact of ART may be particularly intense when the treatment fails and new cycles are needed. Specifically, recent literature shows that repeated ART attempts are associated with recurring frustrated expectations, loss of hope, lower quality of life and an increased risk of depression and/or anxiety (Monti et al., 2015; Moura-Ramos et al., 2016; Agostini et al., 2017, 2018; Molgora et al., 2020). Moreover, previous failed ART cycles may negatively influence the quality of parent-infant interactions (Wang et al., 2014; Allan et al., 2019) and represent an aggravating factor, associated with high infant fussiness and difficulties during free parent-infant interactions (McMahon et al., 1997; Agostini et al., 2020). Treatment type represents another influencing variable to be included in studies about ART parenting, as it associates to the severity of the infertility diagnosis. Specifically, Agostini et al. (2020) showed that infants conceived through ICSI had higher levels of both compulsivity and passivity during interactions with their parents compared to IVF infants.

All these studies well support the psychological burden of ART and suggest that parenting after ART pregnancies may be challenging and emotionally highly demanding (Hammarberg et al., 2008; Ranjbar et al., 2020). Indeed, not only the transition to parenthood regards the welcoming of a baby, but it also represents a profound psychological and emotional experience that brings future mothers and fathers to activate their caregiving system, as well as to adjust to the new parental role (Ammaniti et al., 1992, 2013; Stern, 1995; Ilicali and Fisek, 2004; Slade et al., 2009; Raphael-Leff, 2010).

However, early parenting and psychological processes involved in the transition to parenthood in the context of ART pregnancies have not been investigated enough (Hammarberg et al., 2008), therefore more research in this field is recommended.

The activation of the caregiving system and the development of a parental role require a profound reorganization of personal identity (Ammaniti et al., 1992; Stern, 1995; Slade et al., 2009; Raphael-Leff, 2010). At a deeper level of psychological processes, during the transition to parenthood mental representations of the self and of the baby are strongly activated. Mental representations can be defined as schemes of reality based upon memories, conscious and unconscious fantasies, expectations and perceptions of past experiences, which shape one’s sense of self and interpersonal behavior (Main et al., 1985; Ammaniti et al., 1992; Stern, 1995; Larney et al., 1997). The construct of mental representations, therefore, well describes the women’s psychological states and processes during perinatal period (Ammaniti et al., 1992, 2013; Stern, 1995; Ilicali and Fisek, 2004). Especially in the second and third trimester of pregnancy, maternal representations regarding self-as mother and the baby arise, in parallel with the growth of the fetus, the starting of fetal movements and the activation of the caregiving system (Ammaniti et al., 1992, 2013; Stern, 1995; Ilicali and Fisek, 2004; Slade et al., 2009; Raphael-Leff, 2010). At the same time, women are supposed to rework the relationship with their partner and with their own mother, in order to define their parental attitude (Ammaniti et al., 1992; Vizziello et al., 1993; Stern, 1995; Cohen and Slade, 2000; Dayton et al., 2010). The process of reorganization of parental representations continues also after childbirth when parental representations are generally enriched by the encounter with the baby’s real characteristics.

Therefore, it is recognized that parental mental representations well describe the state of mind regarding parenthood; besides, they play an important role in predicting early parenting styles (Zeanah and Benoit, 1995). In fact, many studies highlight that the characteristics of prenatal maternal representations may influence the emerging interactive behavior with the infant (Fonagy et al., 1991; Ammaniti et al., 1992; Stern, 1995; Zeanah and Benoit, 1995; Flykt et al., 2012).

Based on this evidence, the assessment and investigation of mental representations during the perinatal period is recommended. Particularly, both the content of mental representations and their narrative structure should be taken into account (Cramer, 1989; Stern, 1991; Zeanah et al., 1994; Ammaniti et al., 1995). Ammaniti et al. (1995, 2013) developed the Interview of Maternal Representations During Pregnancy (IRMAG; Ammaniti et al., 1995, 2006) to assess mental representations describing three main types of representations according to how the mother copes with the experience of motherhood and with the forthcoming baby: Integrated/Balanced, Restricted/Disinvested, Not integrated/Ambivalent. The IRMAG interview also includes an adjectives list, built on the model of semantic differentials (Osgood et al., 1957) and concerning contents of mental representations (the baby, the self-as-woman, the partner, the self-as-mother, and the own-mother).

Recent studies showed that the reorganization of parental representations during pregnancy is active in men too (Vreeswijk et al., 2014, 2015), even if paternal representations have been less investigated. Nevertheless, when compared with their female partners, men would tend to show more frequently disengaged representations of their infants (Vreeswijk et al., 2014, 2015).

There is enough evidence that the nature and the quality of parental mental representations may be impaired in presence of specific risk conditions (i.e., psycho-social risk, depressed, or drug-abusing women), where high levels of not integrated maternal representations have been observed, with negative consequences on early parenting skills too (Pajulo et al., 2001; Wendland and Miljkovitch, 2003; Flykt et al., 2012; Ammaniti et al., 2013; Davis et al., 2020).

Interestingly, very little attention has been paid to the investigation of mental representations in the context of parenting following ART. To our knowledge, only the study by Agostini et al. (2009) analyzed the quality of parental mental representations comparing spontaneous conceiving (SC) mothers with ART mothers, showing that ART women had less integrated and more ambivalent representations compared to controls, both during pregnancy vs. 3 months postpartum. Furthermore, a high prevalence of disengaged representations was observed in ART fathers (Agostini et al., 2009). However, that study lacked a sample of SC fathers for more complete comparisons.

Given the emotional challenges related to the transition to parenthood after ART pregnancies, the study of parental representations is useful for the advancement of this field of research and for the improvement of clinical practice. Previous literature underlined how infertility and ART treatments may impair both women’s and men’s affective states, possibly mental representations too, in terms of perceiving themselves as not able to fulfill one’s generative role, with low self-esteem and low self-confidence (Hammarberg et al., 2008; Ladores and Aroian, 2015; Alamin et al., 2020; Ranjbar et al., 2020).

Therefore, the aim of the present study was to investigate the quality of parental mental representations during pregnancy and after delivery in ART parents, in comparison with SC parents. Specifically, we aimed to answer to the following research questions: (1) Do mental representations in the perinatal period differ depending on both conceiving method (ART vs. SC), parental role (mother vs. father) and time of assessment (at 3rd trimester of pregnancy vs. 3 months postpartum)? (2) Are the characteristics of mental representations in ART parents influenced by variables pertaining ART treatment, such as cause of infertility, presence of previous ART attempts, and treatment type?

## Materials and Methods

### Participants

Seventeen couples who conceived through ART (*M*_*age*_ = 38.6, SD = 5.7) and 25 SC couples (*M*_*age*_ = 33.5, SD = 4.7) were recruited at the Santa Maria Nuova Hospital in Reggio Emilia (Italy). A retrospective examination of the adequacy of the number of participants for repeated measure ANOVA was run through the software G^∗^Power 3.1. Repeated measure ANOVAs with within-between interactions, two assessment points and four groups (i.e., ART vs. SC and mothers vs. fathers) were considered in order to calculate the achieved power. A total sample size of 84 participants reached a power of 0.97 which is conventionally deemed to be satisfactory (Faul et al., 2009).

Inclusion criteria for ART and SC couples were: good understanding of the Italian language, absence of any major complications during pregnancy and at childbirth (including preterm births), neonatal or maternal severe disease in the perinatal period. Specific additional inclusion criteria for the ART group were: maternal age lower than 44 years (in accordance with the Hospital guidelines for ART treatments), and having a successful IVF/ICSI cycle using fresh and ejaculated sperm.

### Procedure

This study was part of a wider longitudinal study, involving both ART and SC couples from 20 gestational weeks up to 10 months postpartum. In this paper, we only present data regarding 32 weeks of pregnancy and 3 months after childbirth.

Couples were contacted by a psychologist of the Hospital in occasion of the morphological ultrasound visit, at around 20 gestational weeks. At enrollment, participants were given detailed information on the study aims and protocol, and were asked to sign an informed consent form. Participation was voluntary and anonymous. At both 32 gestational weeks (T1) and 3 months after birth (T2), all couples who agreed to participate received an envelope containing a questionnaire booklet, for the assessment of parental representations (through the Semantic Differential) and depression, anxiety, prenatal attachment, social support through other instruments; the couples also received an additional envelope for returning the material. The study was conducted according to the Helsinki Declaration, though it was not submitted to the Ethics committee of the Hospital because at the time of data collection the Italian law for non-interventional study did not require it.

### Measures

#### Demographic and Obstetric Variables

A questionnaire was created to collect parental demographic characteristics (e.g., age, level of education, current employment) and obstetric variables (e.g., number of previous pregnancies and deliveries). Additionally, ART participants were asked to provide information about their ART history (number of previous ART attempts, treatment type, and cause of infertility).

#### Parental Representations

The Semantic Differential of the IRMAG (Interview of Maternal Representations During Pregnancy; Ammaniti et al., 1992, 1995, 2006) and of the IRPAG (Interview of Paternal Representations During Pregnancy; Ammaniti et al., 2006) was used to assess parental mental representations during pregnancy and after childbirth. This instrument is generally used for research goals as a self-report and independently from the interview (Pajulo et al., 2001, 2004). The semantic differentials of IRMAG have already been used in previous studies including psychosocial and depressive risk (Pajulo et al., 2001; Ammaniti et al., 2013), single mothers (Wendland and Miljkovitch, 2003), drug abusing mothers (Flykt et al., 2012), couples with prenatal diagnosis of fetal anomaly (Giuliani et al., 2014).

The Semantic Differential explores five dimensions of parenthood experiences in terms of mental representations regarding: the child, self-as-woman/man, partner (individual characteristics), self-as-mother/father and own mother/father characteristics (parental characteristics). Each dimension is measured through a list of 17 pairs of opposite adjectives (e.g., self-confident/insecure, calm/anxious, joyful/serious, permissive/authoritarian) placed at one and the other end of a horizontal line (10 cm long), so that respondents are required to mark the point from 0 to 10 that best indicates their description. For each pair of adjectives, a score of 10 corresponds to the more positively-laden adjective. Global scores for each dimension were computed by averaging the scores obtained at the relevant adjective list, so that a higher score corresponds to a more positive representation.

Other specific scores were calculated, according to four areas based on factorial analyses as identified by Ammaniti et al. (1995). For what concerns the representations of individual characteristics (dimensions of child, self-as-woman/man and partner), four areas were calculated: Personal functioning, Interpersonal style, Emotional tendencies, Content of impulses. Regarding parental characteristics (dimensions of self-as-mother/father and own mother/father’s characteristics), the following four areas were considered: Personal functioning, Maternal/paternal role, Maternal/paternal interaction and sensitivity and Emotional tendencies.

### Statistical Analysis

Demographic and obstetric data were compared between ART and SC parents using Pearson’s χ^2^ test and Student’s *t* test for independent samples for nominal and continuous variables, respectively.

To examine mean-level differences between parents who conceived through ART and parents who conceived spontaneously, a series of repeated-measures analyses of variance (ANOVAs) were conducted. Each model included two between-subject variables (conception modality: ART vs. SC; parental role: mother vs. father), and time of assessment (T1 vs. T2) as a within-subject variable. Single ANOVAs were run for every dimension of the semantic differential and the relative four representation areas pertaining either individual (Child, Self, and Partner) and parental (Self-as-mother/father, Own parent) characteristics.

The same analytic strategy was used to explore differences within the sample of ART conceiving parents. The variables parental role (mother vs. father) and time of assessment (T1 vs. T2) were taken into account together with one among the following between-subject factors: presence of previous unsuccessful ART attempts (yes vs. no), treatment type (IVF vs. ICSI), or cause of infertility (female factor vs. male factor).

All statistical analyses were performed using SPSS (version 25) for Windows (IBM, Armonk, NY, United States). In all statistical tests, a *P* value of less than 0.05 was considered significant.

## Results

### Demographic and Obstetric Characteristics

Differences in demographic and obstetric variables between ART and SC parents are shown in Table 1. Overall, all parents were employed and married, and 90% of them was born in Italy. The only statistically significant demographic difference between parents was in age, as mothers and fathers who conceived through ART were older compared to their Spontaneous counterparts [*F*(1,84) = 7.5, *p* < 0.001]. Such result is in line with data coming from both the last report on fertility of the Italian National Institute for Statistics (Istituto Nazionale di Statistica [ISTAT, Italian National Institute for Statistics], 2018; Registro Nazionale sulla Procreazione Medicalmente Assistita [National Assisted Reproduction Registry of Italy], 2017). With regards to obstetric variables, ART babies had a lower weight at birth compared to SC babies (*t* = 2.48, *p* < 0.05).

**TABLE 1:** Main demographic and obstetric characteristics in ART and Spontaneous conceiving (SC) parents.

	ART (*N* = 34)			SC (*N* = 50)			
	Fathers (*N* = 17)	Mothers (*N* = 17)	Total (*N* = 34)	Fathers (*N* = 25)	Mothers (*N* = 25)	Total (*N* = 50)	*p* value
**Demographic characteristics**							
Mean age in years (SD)	39.7 (7.2)	37.5 (3.6)	38.6 (5.7)	34.4 (4.8)	32.7 (4.6)	33.5 (4.7)	0.001
Place of birth, *n* (%)							0.461
Italy	15 (88.2)	15 (88.2)	30 (88.2)	23 (92)	23 (92)	46 (92)	
Abroad	2 (11.8)	2 (11.8)	4 (11.8)	2 (8)	2 (8)	4 (8)	
Level of education, *n* (%)							0.187
Secondary school	3 (17.7)	2 (11.7)	5 (14.7)	5 (20)	2 (8)	7 (14)	
High school	9 (52.9)	7 (41.2)	16 (47.1)	11 (44)	8 (32)	19 (38)	
University	5 (29.4)	8 (47.1)	13 (38.2)	9 (36)	15 (60)	24 (48)	
**Obstetric characteristics**							
Type of delivery, *n* (%)							
Natural childbirth			10 (58.8)			19 (79.2)	0.148
Caesarian section			7 (41.2)			5 (41.7)	
Mean gestational age at birth in weeks (SD)			38.7 (3.2)			39.1 (1.5)	0.065
Mean birth weight in grams (SD)			2999 (0.62)			3406 (.41)	0.018
Sex, *n* (%)							
Male			6 (35.3)			16 (64)	0.067
Female			11 (64.7)			9 (36)	

With respect to ART parents, data regarding infertility history showed that the prevalent cause of infertility was due to a male factor (*n* = 18; 52.9%; e.g., varicocele), followed by a female factor (*n* = 16; 47.1%), related either to women’s age (*n* = 8; 50%; e.g., low AMH values) or endometriosis (*n* = 8; 50%). The majority of our sample (*n* = 22; 64.7%) achieved pregnancy with ICSI, while the remaining couples achieved pregnancy through IVF (*n* = 12; 35.3%). Most participants were at their first ART attempt (*n* = 20; 58.8%), with number of previous ART attempts for the overall sample ranging from 0 to 4 (*M* = 0.88, SD = 1.3).

### Semantic Differentials Dimensions in ART and Spontaneous Conceiving Parents

Detailed presentations of the results from repeated measures ANOVAs on semantic differentials and the four representation areas for individual and parental characteristics are shown in Tables 2, 3, respectively.

**TABLE 2:** Means ± Standard Deviations for each dimension of the Semantic Differentials in ART (*N* = 34) and SC (*N* = 50) parents at 32 gestational weeks and 3 months after delivery.

	ART						SC					
	Fathers		Mothers		Total		Fathers		Mothers		Total	
Dimensions	T1	T2	T1	T2	T1	T2	T1	T2	T1	T2	T1	T2
Child	7.49 ± 1.05	7.47 ± 0.98	7.54 ± 0.89	7.51 ± 0.98	7.52 ± 0.96	7.49 ± 0.97	7.09 ± 0.95	6.89 ± 0.82	7.16 ± 0.81	7.19 ± 0.81	7.12 ± 0.88	7.04 ± 0.83
Self-as-woman/man	7.53 ± 0.97	7.82 ± 0.94	7.25 ± 0.97	7.48 ± 1.43	7.39 ± 0.96	7.65 ± 1.20	7.16 ± 0.87	7.25 ± 0.75	7.31 ± 0.89	7.57 ± 0.98	7.24 ± 0.87	7.41 ± 0.88
Partner	7.75 ± 1.10	7.70 ± 1.09	7.71 ± 1.16	7.70 ± 0.86	7.73 ± 1.11	7.71 ± 0.98	7.10 ± 1.03	7.44 ± 0.98	7.32 ± 0.89	7.70 ± 1.02	7.21 ± 0.96	7.57 ± 1.00
Self-as-parent	7.33 ± 0.90	7.36 ± 0.79	7.10 ± 0.71	7.38 ± 0.65	7.21 ± 0.81	7.37 ± 0.71	6.94 ± 1.34	6.75 ± 0.82	7.11 ± 0.81	7.50 ± 0.81	7.02 ± 1.09	7.13 ± 0.89
Own parent	6.46 ± 1.42	6.61 ± 1.43	6.67 ± 1.48	6.66 ± 1.60	6.57 ± 1.43	6.64 ± 1.50	6.26 ± 1.51	6.12 ± 1.40	6.92 ± 1.41	6.93 ± 1.75	6.60 ± 1.48	6.53 ± 1.62

*ART, assisted reproductive treatments; T1, 32 gestational weeks; T2, 3 months after delivery.*

**TABLE 3:** Means ± Standard Deviations for the four representation areas for individual and parental characteristics in ART (*N* = 34) and SC (*N* = 50) parents at 32 gestational weeks and 3 months after delivery.

	ART						SC					
	Fathers		Mothers		Total		Fathers		Mothers		Total	
	T1	T2	T1	T2	T1	T2	T1	T2	T1	T2	T1	T2
**Individual characteristics**												
Personal functioning												
Child	44.33 ± 7.17	43.16 ± 10.57	42.33 ± 6.50	43.73 ± 5.67	43.33 ± 6.81	43.45 ± 8.36	41.21 ± 6.34	40.42 ± 5.16	41.82 ± 5.54	40.72 ± 4.99	41.51 ± 5.90	40.57 ± 5.03
Self-as-woman/man	42.49 ± 8.85	45.32 ± 5.55	42.87 ± 8.10	42.56 ± 6.79	42.68 ± 8.36	43.94 ± 6.26	41.97 ± 8.37	41.09 ± 6.14	40.70 ± 7.27	42.00 ± 5.92	41.02 ± 7.81	41.55 ± 5.98
Partner	43.98 ± 9.60	46.91 ± 8.25	45.54 ± 7.24	45.97 ± 5.53	44.74 ± 8.44	46.46 ± 6.97	40.94 ± 6.45	42.87 ± 6.47	43.36 ± 6.62	45.09 ± 7.31	42.25 ± 6.58	43.98 ± 6.92
Interpersonal style												
Child	21.86 ± 4.99	24.12 ± 4.11	22.98 ± 4.41	22.76 ± 3.88	22.42 ± 4.67	23.44 ± 3.99	21.54 ± 3.84	20.03 ± 3.59	23.05 ± 4.57	21.14 ± 3.92	22.29 ± 4.25	20.59 ± 3.76
Self-as-woman/man	22.40 ± 4.18	23.18 ± 4.18	24.33 ± 9.54	26.68 ± 17.11	23.37 ± 7.31	24.93 ± 12.39	21.43 ± 4.39	22.72 ± 3.07	23.52 ± 3.58	24.50 ± 3.42	22.47 ± 4.10	23.61 ± 3.34
Partner	23.49 ± 4.46	24.09 ± 4.08	23.71 ± 4.73	22.79 ± 5.94	23.60 ± 4.53	23.46 ± 5.03	22.02 ± 4.17	22.47 ± 3.84	22.83 ± 4.72	23.78 ± 5.90	22.43 ± 4.43	23.13 ± 4.97
Emotional tendencies												
Child	38.33 ± 5.03	36.70 ± 4.63	38.58 ± 5.61	37.99 ± 5.77	38.45 ± 5.25	37.35 ± 5.19	36.97 ± 5.22	35.07 ± 4.97	38.10 ± 4.96	37.49 ± 5.50	37.53 ± 5.07	36.28 ± 5.33
Self-as-woman/man	39.30 ± 5.38	38.31 ± 5.67	36.03 ± 5.37	36.79 ± 5.15	37.66 ± 5.54	37.55 ± 5.39	35.46 ± 5.88	36.70 ± 5.74	37.84 ± 4.30	38.17 ± 4.61	36.65 ± 5.24	37.44 ± 5.02
Partner	37.78 ± 5.13	38.09 ± 5.25	39.93 ± 5.05	39.72 ± 5.22	38.82 ± 5.13	38.88 ± 5.22	34.17 ± 5.71	35.17 ± 4.28	39.97 ± 5.03	40.74 ± 4.74	37.07 ± 6.08	37.95 ± 5.28
Content of impulses												
Child	20.34 ± 3.81	20.77 ± 3.84	21.62 ± 3.52	20.45 ± 4.72	20.98 ± 3.67	20.61 ± 4.24	21.20 ± 4.19	20.85 ± 2.85	19.32 ± 3.07	20.31 ± 3.87	20.21 ± 3.74	20.58 ± 3.38
Self-as-woman/man	23.97 ± 3.30	23.12 ± 4.02	21.86 ± 3.43	21.85 ± 2.80	22.92 ± 3.48	22.48 ± 3.47	21.13 ± 3.30	22.13 ± 3.03	21.11 ± 3.92	22.27 ± 4.47	21.12 ± 3.58	22.20 ± 3.78
Partner	22.66 ± 3.90	21.72 ± 4.10	23.96 ± 3.44	22.78 ± 2.47	23.39 ± 3.67	22.23 ± 3.40	21.56 ± 5.30	22.30 ± 4.43	21.05 ± 4.02	21.99 ± 3.63	21.30 ± 4.66	22.14 ± 4.01
**Parental characteristics**												
Emotional tendencies												
Self-as-parent	23.79 ± 3.37	23.67 ± 3.26	23.22 ± 3.35	23.05 ± 4.28	23.51 ± 3.32	23.36 ± 3.75	22.13 ± 3.22	22.72 ± 2.88	23.76 ± 3.27	25.60 ± 3.33	22.96 ± 3.31	24.19 ± 3.41
Own parent	23.28 ± 4.71	22.67 ± 4.68	20.01 ± 5.34	19.76 ± 6.18	21.54 ± 5.22	21.17 ± 5.62	19.10 ± 6.49	19.87 ± 5.08	21.35 ± 5.55	21.05 ± 6.64	20.22 ± 6.08	20.46 ± 5.88
Personal functioning												
Self-as-parent	36.89 ± 5.95	36.62 ± 6.65	35.61 ± 4.15	37.69 ± 4.12	36.25 ± 5.09	37.16 ± 4.90	32.89 ± 5.89	32.45 ± 4.41	35.34 ± 6.93	38.47 ± 5.54	34.14 ± 6.49	35.53 ± 5.82
Own parent	32.49 ± 9.36	33.37 ± 11.52	34.04 ± 8.92	33.66 ± 9.90	33.29 ± 9.03	33.52 ± 10.55	29.75 ± 8.95	28.32 ± 8.39	35.91 ± 10.48	36.02 ± 9.48	32.83 ± 10.13	32.17 ± 9.67
Parental role												
Self-as-parent	14.59 ± 3.10	15.45 ± 3.24	15.22 ± 2.27	16.11 ± 2.85	14.91 ± 2.91	15.78 ± 3.03	14.16 ± 2.56	15.05 ± 2.98	14.85 ± 2.87	16.47 ± 2.15	14.52 ± 2.72	15.77 ± 2.66
Own parent	14.27 ± 3.65	14.58 ± 4.11	14.34 ± 3.95	13.76 ± 4.30	14.31 ± 3.75	14.16 ± 4.17	12.99 ± 3.75	14.31 ± 3.65	14.50 ± 3.75	14.28 ± 4.87	13.74 ± 3.79	14.29 ± 4.26
Parental interaction and sensitivity												
Self-as-parent	35.35 ± 5.38	35.94 ± 5.63	34.63 ± 3.54	35.50 ± 4.18	34.99 ± 4.50	35.72 ± 4.89	32.43 ± 5.27	31.56 ± 4.60	34.43 ± 5.88	35.49 ± 5.34	33.45 ± 5.62	33.57 ± 5.32
Own parent	31.72 ± 9.54	33.03 ± 7.65	33.71 ± 8.25	33.16 ± 8.71	32.74 ± 8.81	33.10 ± 8.09	31.19 ± 8.73	30.57 ± 7.83	35.14 ± 6.55	35.42 ± 10.70	33.17 ± 7.89	33.00 ± 9.59

*ART, assisted reproductive treatments; T1 = 32 gestational weeks; T2 = 3 months after delivery.*

#### Child

Results on the scores of the Child dimension for ART and Spontaneous parents at T1 and T2 showed a main effect of the variable conception modality [*F*(1,80) = 6.01; *p* < 0.05; partial η^2^ = 0.07], while no parental role [*F*(1,80) = 0.44; *p* = 0.51; partial η^2^ = 0.01] nor time-point effect [*F*(1,80) = 0.28; *p* = 0.60; partial η^2^ = 0.00] and no interaction effects (all *p*s = n.s.) were found. ART parents had overall significantly higher (i.e., more positive representations; *M* = 7.51, SD = 0.96, and *M* = 7.50, SD = 0.97, at T1 and T2, respectively) scores than SC parents (*M* = 7.12, SD = 0.88, and *M* = 7.04, SD = 0.83, at T1 and T2, respectively) on the Child dimension irrespectively of parental role.

With respect to the four representations areas, the only significant result was obtained for Interpersonal Style where an interaction effect time of assessment conception modality [*F*(1,80) = 6.65; *p* < 0.05; partial η^2^ = 0.08] was found: scores for ART parents increased from T1 to T2, while those for SC parents decreased (Table 3).

#### Self-as-Woman/Man

Results on the scores of the Self-as-woman/man dimension for ART and SC parents showed a main effect of the variable time of assessment [*F*(1,80) = 4.90; *p* < 0.05; partial η^2^ = 0.06], while no conception modality [*F*(1,80) = 1.06; *p* = 0.31; partial η^2^ = 0.01] nor parental role [*F*(1,80) = 0.03; *p* = 0.85; partial η^2^ = 0.00], and no interaction effects were found (all *p*s = n.s.). Particularly, for each sub-group there was a significant improvement (i.e., more positive representations) of the representation of the Self from T1 to T2 (*M* = 7.39, SD = 0.96 at T1, and *M* = 7.65, SD = 1.20 at T2, for ART parents; *M* = 7.24, SD = 0.87 at T1, and *M* = 7.41, SD = 0.88 at T2, for SC parents).

With respect to the four representations, a significant interaction assessment × conceiving method × parental role [*F*(1,80) = 4.32; *p* < 0.05; partial η^2^ = 0.05] was obtained for Personal Functioning: Table 3 shows that while for ART fathers and SC mothers scores at this dimension increased from T1 to T2, for ART mothers and SC fathers they remained almost unvaried. Additionally, a significant interaction conception modality × parental role [*F*(1,80) = 4.33; *p* < 0.05; partial η^2^ = 0.05] was found for Emotional Tendencies; while in the case of ART parents, fathers had higher scores compared to mothers ad this dimension, the opposite pattern could be observed for SC couples. Regarding Interpersonal style, a main effect of parental role [*F*(1,80) = 4.10; *p* < 0.05; partial η^2^ = 0.05] indicated higher scores for mothers compared to fathers, irrespectively of conception modality. No significant result for Content of Impulses was observed (all *p*s = n.s.).

#### Partner

Results on the scores of the Partner-dimension showed no main significant effects, neither for the variable conception modality [*F*(1,80) = 2.51; *p* = 0.11; partial η^2^ = 0.03], nor for parental role [*F*(1,80) = 0.30; *p* = 0.58; partial η^2^ = 0.00], nor for time of assessment [*F*(1,80) = 3.67; *p* = 0.06; partial η^2^ = 0.04]. Only an interaction effect of the variable time of assessment × conception modality was found [*F*(1,80) = 4.41; *p* < 0.05; partial η^2^ = 0.06]. As depicted in Table 3, while for ART parents the means and standard deviations at this dimension remained almost stable over time, for SC parents there was an improvement of the scores (i.e., more positive representations) from T1 to T2.

Regarding the four representations for this dimension, a main effect of assessment [*F*(1,80) = 6.46; *p* < 0.08; partial η^2^ = 0.05] was obtained for Personal Functioning, with overall scores improving from T1 to T2 irrespectively of parental role and conception modality. Yet, a main effect of parental role was found for both Emotional Tendencies [*F*(1,80) = 14.85; *p* < 0.001; partial η^2^ = 0.16] and Interpersonal Style [*F*(1,80) = 4.10; *p* < 0.05; partial η^2^ = 0.05]: while in the first case mothers reported better representations of their partners compared to fathers, irrespectively of conception modality, the opposite pattern was observed for Interpersonal Style. No significant results were detected for Content of Impulses (all *p*s = n.s.).

#### Self-as-Mother/Father

Differences on the Self-as-parent dimension scores for ART and SC parents were non-significant for all the variables included in the model, namely conception modality [*F*(1,80) = 1.75; *p* = 0.19; partial η^2^ = 0.02], parental role [*F*(1,80) = 1.16; *p* = 0.28; partial η^2^ = 0.02], and time of assessment [*F*(1,80) = 1.33; *p* = 0.25; partial η^2^ = 0.02], as well as for their interactions (all *p*s = n.s.).

With respect to the four representations areas for this dimension, a significant interaction effect assessment × parental role [*F*(1,80) = 6.12; *p* < 0.05; partial η^2^ = 0.07] was obtained for Personal role, indicating an increase at the scores for this dimension from T1 to T2 for mothers only, irrespectively of conception modality. Additionally, an interaction effect conception modality × parental role [*F*(1,80) = 4.79; *p* < 0.05; partial η^2^ = 0.06] was found for Emotional Tendencies, with SC fathers reporting the lowest scores compared to SC mothers and ART mothers and fathers (see Table 3). A main effect of assessment [*F*(1,80) = 9.15; *p* < 0.01; partial η^2^ = 0.11] resulted for Parental role and showing an increase at this dimension from T1 to T2 for all parents, irrespectively of conception modality. Last, a main effect of conception modality [*F*(1,80) = 4.27; *p* < 0.05; partial η^2^ = 0.05] was found for the area Parental Interaction and Sensitivity, showing significantly higher scores for ART compared to SC parents irrespectively of parental role.

#### Own Parent (Mother/Father)

No significant differences emerged for this dimension between ART and SC parents depending on conception modality [*F*(1,80) = 0.02; *p* = 0.88; partial η^2^ = 0.00], parental role [*F*(1,80) = 1.78; *p* = 0.19; partial η^2^ = 0.02], time of assessment [*F*(1,80) = 0.01; *p* = 0.98; partial η^2^ = 0.00], and their interactions (all *p*s = n.s.).

With respect to the four representation areas, no significant main and interaction effects were detected (all *p*s = n.s.).

### Semantic Differential Dimensions Within the Sample of ART Conceiving Parents

Table 4 presents means and standard deviations for each of the ANOVA models testing the differences from 32 gestational weeks to 3 months after delivery within the sample of ART conceiving parents. Overall, all tested models did not show significant effects (all *p*s = n.s.) for the variables time of assessment (T1 vs. T2) and parental role (mother vs. father).

**TABLE 4:** Means ± Standard Deviations for each dimension of the Semantic Differential in ART conceiving parents at 32 gestational weeks and 3 months after delivery by infertility cause, previous ART attempts, and treatment type.

	Infertility cause				Previous ART attempts				Treatment type			
	Male factor		Female factor		Yes		No		IVF		ICSI	
Dimensions	T1	T2	T1	T2	T1	T2	T1	T2	T1	T2	T1	T2
**Child**												
Fathers	7.41 ± 0.91	7.59 ± 0.91	7.43 ± 1.25	7.17 ± 1.09	7.14 ± 1.05	7.07 ± 0.97	7.81 ± 1.00	7.83 ± 0.90	7.50 ± 1.08	7.79 ± 0.98	7.39 ± 1.07	7.24 ± 0.98
Mothers	7.33 ± 0.74	7.58 ± 0.95	7.58 ± 0.92	7.30 ± 1.10	7.40 ± 0.90	7.38 ± 1.16	7.67 ± 0.92	7.62 ± 0.85	7.96 ± 0.83	7.50 ± 1.19	7.20 ± 0.71	7.44 ± 0.96
Total	7.37 ± 0.81	7.58 ± 0.91	7.50 ± 1.06	7.23 ± 1.05	7.27 ± 0.95	7.22 ± 1.04	7.74 ± 0.93	7.72 ± 0.85	7.72 ± 0.94	7.64 ± 1.03	7.30 ± 0.89	7.34 ± 0.95
**Self-as-woman/man**												
Fathers	7.27 ± 0.85	7.72 ± 0.94	7.70 ± 1.08	7.89 ± 1.04	7.51 ± 0.84	7.56 ± 1.02	7.54 ± 1.12	8.05 ± 0.84	7.62 ± 1.11	8.21 ± 0.89	7.38 ± 0.92	7.60 ± 0.96
Mothers	6.94 ± 0.63	7.02 ± 1.03	7.35 ± 1.03	7.90 ± 1.80	7.15 ± 0.77	7.92 ± 1.63	7.34 ± 1.15	7.09 ± 1.18	7.70 ± 1.02	8.44 ± 1.89	6.86 ± 0.59	6.93 ± 0.93
Total	7.10 ± 0.75	7.37 ± 1.02	7.52 ± 1.03	7.89 ± 1.41	7.33 ± 0.81	7.74 ± 1.33	7.44 ± 1.11	7.57 ± 1.11	7.66 ± 1.01	8.33 ± 1.40	7.11 ± 0.80	7.27 ± 0.99
**Partner**												
Fathers	7.55 ± 0.84	7.64 ± 1.06	7.75 ± 1.29	7.55 ± 1.12	7.47 ± 0.64	7.41 ± 1.15	8.00 ± 1.38	7.96 ± 1.05	7.77 ± 1.49	7.87 ± 1.32	7.57 ± 0.82	7.47 ± 0.95
Mothers	7.26 ± 1.28	7.70 ± 1.01	7.96 ± 0.73	7.84 ± 0.80	7.70 ± 0.85	7.47 ± 0.78	7.73 ± 1.41	7.93 ± 0.92	8.51 ± 0.64	8.06 ± 0.55	7.31 ± 1.17	7.61 ± 1.01
Total	7.42 ± 1.04	7.67 ± 1.01	7.85 ± 1.01	7.69 ± 0.95	7.57 ± 0.72	7.44 ± 0.96	7.87 ± 1.36	7.95 ± 0.96	7.96 ± 1.10	7.97 ± 0.96	7.45 ± 0.98	7.54 ± 0.95
**Self-as-parent**												
Fathers	7.40 ± 0.78	7.49 ± 0.69	7.08 ± 1.03	7.23 ± 0.99	7.60 ± 0.96	7.42 ± 0.90	7.08 ± 0.81	7.32 ± 0.73	6.99 ± 0.99	7.42 ± 0.95	7.38 ± 0.84	7.35 ± 0.79
Mothers	6.73 ± 0.61	7.30 ± 0.72	7.43 ± 0.60	7.36 ± 0.54	7.30 ± 0.65	7.34 ± 0.75	6.92 ± 0.76	7.42 ± 0.60	7.30 ± 0.82	7.18 ± 0.52	6.92 ± 0.62	7.39 ± 0.69
Total	7.06 ± 0.76	7.39 ± 0.69	7.26 ± 0.83	7.29 ± 0.77	7.45 ± 0.81	7.38 ± 0.80	7.01 ± 0.77	7.37 ± 0.65	7.15 ± 0.87	7.30 ± 0.73	7.50 ± 0.76	7.37 ± 0.72
**Own parent**												
Fathers	6.67 ± 1.19	6.94 ± 1.12	6.03 ± 1.66	6.01 ± 1.61	6.93 ± 1.65	7.07 ± 1.58	6.10 ± 1.17	6.26 ± 1.27	6.42 ± 1.16	6.48 ± 1.23	6.35 ± 1.58	6.51 ± 1.55
Mothers	6.23 ± 1.59	6.86 ± 1.74	7.01 ± 1.26	6.19 ± 1.44	6.71 ± 1.67	6.17 ± 1.90	6.64 ± 1.39	7.11 ± 1.22	7.33 ± 1.16	6.38 ± 1.57	6.23 ± 1.51	6.65 ± 1.68
Total	6.44 ± 1.39	6.90 ± 1.43	6.52 ± 1.51	6.10 ± 1.48	6.82 ± 1.61	6.59 ± 1.76	6.37 ± 1.28	6.89 ± 1.28	6.87 ± 1.19	6.43 ± 1.33	6.28 ± 1.51	6.59 ± 1.58

*ART, assisted reproductive treatments; T1 = 32 gestational weeks; T2 = 3 months after delivery; IVF, in vitro fertilization; ICSI, intracytoplasmic sperm injection.*

With regards to the variable cause of infertility (female factor vs. male factor), only a significant interaction time of assessment × cause of infertility emerged on the dimension Own Parent [*F*(1,27) = 5.55; *p* < 0.05; partial η^2^ = 0.17]: scores improved from T1 to T2 for parents with an infertility diagnosis due to male factors, while the opposite pattern emerged for those parents with an infertility diagnosis due to female factors (Table 4). In all other dimensions no significant differences were observed (all *p*s = n.s.).

For what concerns the variable previous ART attempts (yes vs. no), a significant interaction effect parental role × time of assessment × previous ART attempt emerged on the Self-as-woman/man dimension [*F*(1,27) = 4.20; *p* < 0.05; partial η^2^ = 0.12]. Specifically, an improvement from T1 to T2 was observed in fathers with no previous ART attempts, while the same pattern was observed only in mothers who already had previous ART attempts. No significant differences emerged on other dimensions (all *p*s = n.s.).

When the variable treatment type (IVF vs. ICSI) was considered, a main effect emerged on the Self-as-woman/man dimension [*F*(1,28) = 6.31; *p* < 0.05; partial η^2^ = 0.18], revealing more positive representations for those parents who conceived with IVF compared to ICSI, irrespectively of parental role and time of assessment (*M* = 7.12, SD = 0.80 at T1, and *M* = 7.27, SD = 0.99 at T2, for ICSI; *M* = 7.66, SD = 1.01 at T1, and *M* = 8.33, SD = 1.40 at T2, for IVF). No significant effect on any other dimensions emerged (all *p*s = n.s.).

## Discussion

The main aim of the present study was to deepen the knowledge on the transition to parenthood for infertile parents who underwent ART in order to conceive, specifically investigating the characteristics of parental representations.

Despite the psychological burden of infertility and ART (Hammarberg et al., 2008), little is known about the psychological experienced by infertile couples transitioning to parenthood in terms of mental representations about themselves as parents and their baby. Parental representations are predictive for the quality of early parenting behaviors (Zeanah and Benoit, 1995), therefore their investigation in the context of ART parenthood may have potential practical implications for the prevention and treatment of the psychological consequences of conceiving after infertility.

With regards to our first research question, present findings show that the conceiving method had a significant effect on the global representation of the child, but not on the other four dimensions of the Semantic Differential. Indeed, ART parents had more positive representations of their child compared to SC parents, irrespectively of parental role and time of assessment (before or after birth). In previous literature, Pajulo et al. (2004) found that representations of the child were more positive in the case of planned pregnancy, suggesting that these parents were somehow more prepared for the changes required by the arrival of the baby. In our case, ART conception usually occurs after several emotional challenges related to infertility and after a long period of attempts of conceiving through ART (Hammarberg et al., 2008; Flykt et al., 2011); our result may be placed along the lines of a possible and natural mechanism of idealization of parenthood and of the long-awaited child. Previous literature questioned if overly positive representations may act as positive or risk factors for parenting; they could reflect a tendency to maintain inflexible views of the child, which are difficult to change (Flykt et al., 2012), as observed in studies on high-risk samples (Mazzoni, 1992; Ammaniti et al., 1995), or a tendency to be more sensitive and attuned to the baby’s needs, as shown by the good quality observed in ART mother-infant interactions (Tallandini et al., 2012). More longitudinal studies are needed to clarify this issue. The high scores at Child dimension especially emerged in Interpersonal style area, where ART parents’ representations improved from pregnancy to postpartum, while those of SC parents decreased. Because this scale included item such as acceptance, sociability and independency, it could reveal how ART parents further improve their representation of the child after delivery, while SC parents would probably express more the need to adjust to the baby’s arrival.

For what pertains Self-as-woman/man dimension, we found a significant improvement in the passage from pregnancy to 3 months after childbirth, irrespectively of conception modality. Overall, the birth of the baby seems to enrich the positive representation about oneself (Ammaniti et al., 1992; Ilicali and Fisek, 2004), and this evidence may be in line with a possible cultural mandate and the resulting expectation that adult women and men should become parent (Langher et al., 2019). Despite this improvement was observed in all parents, scores at Personal functioning area significantly increased from pregnancy to postpartum period only for ART fathers and SC mothers. The improvement in SC mothers during postpartum period is in line with the literature underlining how the childbirth and the presence of the baby are generally rewarding for the mothers, giving confirmation of their adequacy (Ammaniti et al., 1992; Stern, 1995). This increase did not emerge for ART mothers, because their scores were already high since pregnancy; they probably felt themselves as adequate since the conception, perceived as a success after infertility diagnosis (Hammarberg et al., 2008). For what concern fathers, some specific considerations could be given. Indeed, if we refer to the gender attitude toward parenting in SC pregnancies, men are expected more to provide physical support to the infant and to the partner, being also more oriented to the larger family context, while women are more responsible for affective and emotional caregiving (Winnicott, 1958; Russell et al., 1998). This explanation is consistent with the low scores obtained by SC fathers in Personal functioning both in pregnancy and in postpartum period. Moreover, it could also explain why SC fathers represented themselves with low scores in Emotional tendencies area.

Interestingly, ART fathers showed both an increase in Personal functioning and constant high scores at Emotional tendencies. The more active role played in trying to achieve a conception through ART and the long waiting for a child could explain the higher level of personal involvement since pregnancy (El Kissi et al., 2013; Monti et al., 2015).

Taken together, these results suggested that the representation of Self-as-woman/man could be different in ART and SC parents, especially for men.

Differences between ART and SC parents also emerged regarding the Partner dimension, where only SC parents showed a significant improvement in overall representations from 32 weeks of pregnancy to 3 months postpartum. According to the literature of couple adjustment during the transition to parenthood, results suggest that positive changes in the representations of the partner are gradually activated and achieved in postpartum period, so that the parental couple jointly adapts to the new parental role, while this transition could be affected by the complexity and emotional challenges of conception achieved through ART attempts (Darwiche et al., 2015).

According to Self-as-parent dimension, the effect of conception modality did not emerge for the overall representations, even if it did show a difference for the Parental Interaction and Sensitivity area: ART parents showed higher scores compared to SC parents, suggesting a better representation of their ability to interact with their baby. This result could be explained considering two elements. First, a tendency to idealize and invest on both the baby and the relationship with her/him, that should return to parent the efforts spent into conceiving (Hammarberg et al., 2008). Second, studies investigating the quality of early interactions between ART parent and infant, by using observative tools, have often shown not enough sensitive and adequate patterns (La Sala et al., 2004; Cairo et al., 2012; Agostini et al., 2020). Therefore, the highly positive representations would not seem supported by the effective quality of interactive patterns and would suggest some difficulties in taking care of the “real” baby (Lier et al., 1995).

Another interesting result about Self-as-parent dimension regarded the Emotional tendencies area: SC fathers got lower scores compared to all other parents (SC mothers, ART mothers, ART fathers), suggesting again in this group a tendency to be less affectively and emotionally involved than other parents, as already emerged for SC fathers in the Emotional tendencies area for the representation of Self-as-man.

Finally, when Own parent dimension was considered, no significant effects emerged. Actually, this dimension is related to a relevant psychological process of the transition to parenthood. Indeed, during pregnancy, the work through one’s childhood experiences requires to women a re-elaboration of the relationship with their own mother (Vizziello et al., 1993; Cohen and Slade, 2000) and, in parallel, in men in relation to their own father: in adequate conditions, this intrapsychic work should lead to accept and recognize being similar to own parents (Cramer, 2000). Conversely, previous studies showed that specific risk conditions (i.e., depression or drug addiction) could interfere with this process, leading future parents to see themselves more negatively and less similar to their own mothers/fathers after the childbirth (Mazzoni, 1992; Ammaniti et al., 1995). In the context of ART, representations of own parental figures could be influenced, because in most cases their own parents were able to conceive naturally. Nevertheless, all these psychological processes of identification and differentiation from own parental figures occur mostly at an unconscious level, therefore it is possible that the Semantic Differential did not detect possible significant effects. Anyway, we got interesting results when we considered specific ART variables (cause of infertility, previous ART attempts, and the treatment type) and their influence on parental representations, according to our second aim of the study.

In particular, a significant effect of the cause of infertility emerged, confirming that the infertility diagnosis (i.e., the role and contribution of female or male factors on infertility) could represent a obstacle for adequate representation of own parents. Nevertheless, while the role of maternal factor on infertility increased after childbirth, the effect of male infertility could permian and intensify in postpartum period, with a worsening of the representation. It is possible that the mothers, through the achievement of a pregnancy and giving physically birth the baby, could retrieve elements of contact with their mothers, promoting an improvement of her representation. Conversely, for men the birth of baby, despite desired, could not be enough to improve their sense of inadequacy due to infertility. However, given the absence of previous literature and the small size of our sample, these considerations should be taken with caution and confirmed by future studies.

With regards to the effects of treatment type and previous ART attempts, we found that both variables were associated to a worse representation of Self-as-woman/man, in line with already existing literature attesting the detrimental effects of these variables on psychological wellbeing (Monti et al., 2015; Moura-Ramos et al., 2016; Agostini et al., 2017, 2018) and quality of parent-infant interactions (Agostini et al., 2020). Specifically, we observed more negative representations of Self-as-woman/man in parents who underwent ICSI when compared to IVF counterparts; this might be related to the fact that, in our sample, ICSI was chosen as reproductive technique in the case of a more severe infertility diagnosis, which in turn might have negatively affected parents’ self-image. Furthermore, a negative effect of previous failed ART attempts emerged, thus suggesting this variable as a potential risk factor for negative psychological outcomes, such as anxiety and depressive symptomatology (Monti et al., 2015; Moura-Ramos et al., 2016; Agostini et al., 2017, 2018; Alamin et al., 2020). Yet, parental representations improved from pregnancy to three postpartum months only in case of mothers with previous ART attempts and of fathers at their first ART cycle. This result should be further explored by future studies, specifically taking into account both parents’ vulnerabilities and resilience that persist ART after failures. Taken together, our results reinforce the knowledge on the role played by variables attesting the severity of infertility over psychological functioning. At the same time, they suggest the relevance of including clinical data on infertility and ART history in future studies on pregnancies after ART. For instance, it would be important to further understand how the diagnosis of infertility as well as its severity may impact individual’s and couples’ transition to parenthood.

Limitations of the present investigation pertain methodological issues, as only one self-report measure was included, analyses were performed over small groups by using comparisons only (ANOVA), and parental psychological wellbeing (e.g., measures of anxiety and depression) was not included. Particularly, the small sample size did not allow more in-depth analyses. For instance, we couldn’t control for the effects of relevant covariates such as age, which was significantly different between ART and SC parents, and the actual number of previous unsuccessful ART attempts, as well as the specific infertility diagnosis (e.g., azoospermia, endometriosis, and premature ovarian failure), for analyses pertaining ART sample only. Given the relevance of these variables, we suggest their inclusion in future studies with larger sample size.

Despite such limitations and the need for caution in generalizing present findings, it is important to stress that the novelty of this study relies on the focus on the longitudinal assessment of parental mental representations in ART and SC parents, both fathers and mothers. Globally, present results did not show relevant differences in mental representations between ART and SC parents. On the one hand, this may suggest that for those couples who successfully conceived after ART, according to the specific clinical characteristics of the sample, the psychological process related to the transition to parenthood may be similar to that of SC parents. On the other hand, our data put a light on some specific differences which should be addressed more in depth by future studies, in order to better identify peculiarities of the process of becoming a parent in the context of ART.

Current literature shows a dearth of published studies focusing on parental mental representations, and this is one among the few recent investigations which explored this issue, especially within the context of pregnancies after ART. It is worth mentioning that the only other published study on the same topic (Agostini et al., 2009) showed some different results. This could be explained to a certain extent by methodological issues; indeed, Agostini et al. analyzed parental representations by using the semi-structured interview, with the aim of identifying the type of parental representations; however, they did not include in their investigation neither a group of SC fathers nor the effects of clinical variables pertaining ART treatment.

Given the potential psychological consequences of infertility and ART, and considering the emotional challenges related to the transition to parenthood, further studies reinforcing present findings are recommended, as the assessment of parental representations is relevant for a more complete understanding of psychological processes in both mothers and fathers and may help clinicians in tailoring more personalized support to ART couples.

## Data Availability Statement

The datasets presented in this article are not readily available because data refer to a clinical population and therefore are confidential. Requests to access the datasets should be directed to FAn, federica.andrei2@unibo.it.

## Ethics Statement

Ethical review and approval was not required for the study on human participants in accordance with the local legislation and institutional requirements. The patients/participants provided their written informed consent to participate in this study.

## Author Contributions

MP, ET, SS, MV, and LA contributed to prepare the study design, supervised data collection, and the research team. FAn performed statistical analysis, prepared the tables, and contributed to the writing of all the sections of the manuscript. EN contributed to the writing of all the sections of the manuscript. FAg prepared the study design, supervised all the phases of the research study, and contributed to the writing of all the sections of the manuscript. All authors reviewed and approved the manuscript for publication.

## Conflict of Interest

The authors declare that the research was conducted in the absence of any commercial or financial relationships that could be construed as a potential conflict of interest.

## References

[B1] Agostini F. Andrei F. Neri E. Trombini E. Nuccini F. Villani M. T. (2020). Characteristics of early mother-infant and father-infant interactions: a comparison between assisted reproductive technology and spontaneous conceiving parents. *Int. J. Environ. Res. Public Health* 17:8215. 10.3390/ijerph17218215 33172139 PMC7664381

[B2] Agostini F. Monti F. Andrei F. Paterlini M. Palomba S. La Sala G. B. (2017). Assisted reproductive technology treatments and quality of life: a longitudinal study among subfertile women and men. *J. Assist. Reprod. Genet.* 34 1307–1315. 10.1007/s10815-017-1000-9 28733802 PMC5633563

[B3] Agostini F. Monti F. Fagandini P. Duncan De Pascalis L. L. La Sala G. B. Blickstein I. (2009). Parental mental representations during late pregnancy and early parenthood following assisted reproductive technology. *J. Perinat. Med.* 37 320–327. 10.1515/JPM.2009.062 19290854

[B4] Agostini F. Monti F. Paterlini M. Andrei F. Palomba S. La Sala G. B. (2018). Effect of the previous reproductive outcomes in subfertile women after in vitro fertilization (IVF) and/or intracytoplasmic sperm injection (ICSI) treatments on perinatal anxious and depressive symptomatology. *J. Psychos. Obstetr. Gynecol.* 39 29–37. 10.1080/0167482x.2017.1286474 28635535

[B5] Alamin S. Allahyari T. Ghorbani B. Sadeghitabar A. Karami M. T. (2020). Failure in identity building as the main challenge of infertility: a qualitative study. *J. Reprod. Infertility* 21 49–58. PMC7048687 32175265

[B6] Allan H. T. van den Akker O. Culley L. Mounce G. Odelius A. Symon A. (2019). An integrative literature review of psychosocial factors in the transition to parenthood following non-donor-assisted reproduction compared with spontaneously conceiving couples. *Hum. Fertil. (Camb)* 1–18. 10.1080/14647273.2019.1640901 31328586

[B7] Ammaniti M. Baumgartner E. Candelori C. Perucchini P. Pola M. Tambelli R. (1992). Representations and narratives during pregnancy. *Infant Ment. Health J.* 13 167–182. 10.1002/1097-0355(199223)13:2<167::AID-IMHJ2280130207<3.0.CO;2-M

[B8] Ammaniti M. Candelori C. Pola M. Tambelli R. (1995). *Maternità e Gravidanza.* Milano: Raffaello Cortina Editore.

[B9] Ammaniti M. Tambelli R. Odorisio F. (2006). Intervista clinica per lo studio delle rappresentazioni paterne in gravidanza: IRPAG. *Età Evol.* 85 30–40.

[B10] Ammaniti M. Tambelli R. Odorisio F. (2013). Exploring maternal representations during pregnancy in normal and at-risk samples: the use of the interview of maternal representations during pregnancy. *Infant Ment. Health J.* 34 1–10. 10.1002/imhj.21357

[B11] Cairo S. Darwiche J. Tissot H. Favez N. Germond M. Guex P. (2012). Family interactions in IVF families: change over the transition to parenthood. *J. Reprod. Infant Psychol.* 30 5–20. 10.1080/02646838.2012.669830

[B12] Cohen L. Slade A. (2000). “The psychology and psychopathology of pregnancy: reorganization and transformation,” in *Handbook of Infant Mental Health*, 2nd Edn, ed. Zeanah C. H. (New York, NY: Guilford Press), 20–36.

[B13] Cramer B. (1989). *Profession Bebè.* Paris: Calman-Levy.

[B14] Cramer B. (2000). *Cosa Diventeranno I Nostri Bambini*? Milano: Raffaello Cortina Editore.

[B15] Darwiche J. Favez N. Simonelli A. Antonietti J. P. Frascarolo F. (2015). Prenatal coparenting alliance and marital satisfaction when pregnancy occurs after assisted reproductive technologies or spontaneously. *Fam. Relat.* 64 534–546. 10.1111/fare.12131

[B16] Davis J. A. G. Alto M. E. Oshri A. Rogosch F. Cicchetti D. Toth S. L. (2020). The effect of maternal depression on mental representations and child negative affect. *J. Affect. Disord.* 261 9–20. 10.1016/j.jad.2019.09.073 31600590 PMC6936600

[B17] Dayton C. J. Levendosky A. A. Davidson W. S. Bogat G. A. (2010). The child as held in the mind of the mother: the influence of prenatal maternal representations on parenting behaviors. *Infant Ment. Health J.* 31 220–241. 10.1002/imhj.20253 28543330

[B18] El Kissi Y. Romdhane A. B. Hidar S. Bannour S. Ayoubi Idrissi K. Khairi H. (2013). General psychopathology, anxiety, depression and self-esteem in couples undergoing infertility treatment: a comparative study between men and women. *Eur. J. Obstet. Gynecol. Reprod. Biol.* 167 185–189. 10.1016/j.ejogrb.2012.12.014 23298895

[B19] Fassino S. Pierò A. Boggio S. Piccioni V. Garzaro L. (2002). Anxiety, depression and anger suppression in infertile couples: a controlled study. *Hum. Reprod.* 17 2986–2994. 10.1093/humrep/17.11.2986 12407062

[B20] Faul F. Erdfelder E. Buchner A. Lang A. G. (2009). Statistical power analyses using G^∗^ Power 3.1: tests for correlation and regression analyses. *Behav. Res. Methods* 41 1149–1160. 10.3758/brm.41.4.1149 19897823

[B21] Ferraretti A. P. Nygren K. Andersen A. N. de Mouzon J. Kupka M. Calhaz-Jorge C. (2017). Trends over 15 years in ART in Europe: an analysis of 6 million cycles. *Hum. Reprod. Open* 2017:hox012. 10.1093/hropen/hox012 31486803 PMC6276702

[B22] Flykt M. Lindblom J. Punamäki R. L. Poikkeus P. Repokari L. Unkila-Kallio L. (2011). Prenatal expectations in transition to parenthood: former infertility and family dynamic considerations. *Couple Fam. Psychol. Res. Pract.* 1 31–44. 10.1037/2160-4096.1.s.31 20001136

[B23] Flykt M. Punamäki R. -L. Belt R. Biringen Z. Salo S. Posa T. (2012). Maternal representations and emotional availability among drug-abusing and nonusing mothers and their infants. *Infant Ment. Health J.* 33 123–138. 10.1002/imhj.21313 28520099

[B24] Fonagy P. Steele H. Steele M. (1991). Maternal representations of attachment during pregnancy predict the organization of infant-mother attachment at one year of age. *Child Dev.* 62 891–905. 10.1111/j.1467-8624.1991.tb01578.x 1756665

[B25] Giuliani R. Tripani A. Pellizzoni S. Clarici A. Lonciari I. D’Ottavio G. Schleef J. (2014). Pregnancy and postpartum following a prenatal diagnosis of fetal thoracoabdominal malformation: the parental perspective. *J. Pediatr. Surg.* 49 353–358. 10.1016/j.jpedsurg.2013.07.025 24528985

[B26] Hammarberg K. Fisher J. R. W. Wynter K. H. (2008). Psychological and social aspects of pregnancy, childbirth and early parenting after assisted conception: a systematic review. *Hum. Reprod. Update* 14 395–414. 10.1093/humupd/dmn030 18653674

[B27] Hjelmstedt A. Collins A. (2008). Psychological functioning and predictors of father-infant relationship in IVF fathers and controls. *Scand. J. Caring Sci.* 22 72–78. 10.1111/j.1471-6712.2007.00537.x 18269425

[B28] Ilicali E. T. Fisek G. O. (2004). Maternal representations during pregnancy and early motherhood. *Infant Ment. Health J.* 25 16–27. 10.1002/imhj.10082

[B29] Istituto Nazionale di Statistica [ISTAT, Italian National Institute for Statistics] (2018). *Report. Natalità e Fecondità Della Popolazione Residente (anno 2017).* Available online at: https://www.istat.it/it/files/2019/11/Report_natalità_anno2018_def.pdf (accessed February 1, 2021).

[B30] La Sala G. B. Gallinelli A. Fagandini P. Bevolo P. Landini A. Ballabeni A. (2004). Developmental outcomes at one and two years of children conceived by intracytoplasmic sperm injection. *Int. J. Fertility Women’s Med.* 49 113–119. 15303312

[B31] Ladores S. Aroian K. (2015). First-time mothers with a history of infertility: their internalized pressure to breastfeed. *J. Hum. Lactation* 31 504–510. 10.1177/0890334415585511 25944646

[B32] Langher V. Fedele F. Caputo A. Marchini F. Aragona C. (2019). Extreme desire for motherhood: analysis of narratives from women undergoing assisted reproductive technology (ART). *Eur. J. Psychol.* 15 292–311. 10.5964/ejop.v15i2.1736 33574956 PMC7871747

[B33] Larney B. Cousens P. Nunn K. (1997). Maternal representations reassessed. *Clin. Child Psychol. Psychiatry* 2 125–144.

[B34] Lier L. Gammeltoft M. Knudsen I. J. (1995). Early mother-child relationship. The Copenhagen model of early preventive intervention towards mother-infant relationship disturbances. *Arctic Med. Res.* 54 15–23. 7639899

[B35] Main M. Kaplan N. Cassidy J. (1985). “Security in infancy, childhood and adulthood: a move to the level of representation,” in *Growing Points of Attachment Theory and Research. Monographs of the Society for Research in Child Development*, Vol. 50 eds Bretherton I. Waters E. (Chicago, IL: University of Chicago press), 66–104. 10.2307/3333827

[B36] Mascarenhas M. N. Flaxman S. R. Boerma T. Vanderpoel S. Stevens G. A. (2012). National, regional, and global trends in infertility prevalence since 1990: a systematic analysis of 277 health surveys. *PLoS Med.* 9:e1001356. 10.1371/journal.pmed.1001356 23271957 PMC3525527

[B37] Mazzoni S. (1992). “Tossicomania e gravidanza,” in *La gravidanza tra Fantasia e Realtà*, ed. Ammaniti M. (Roma: Il Pensiero Scientifico), 217–235.

[B38] McLachlan R. I. O’Bryan M. K. (2010). Clinical Review#: state of the art for genetic testing of infertile men. *J. Clin. Endocrinol. Metab.* 95 1013–1024. 10.1210/jc.2009-1925 20089613

[B39] McMahon C. A. Ungerer J. A. Tennant C. Saunders D. (1997). Psychosocial adjustment and the quality of the mother-child relationship at four months postpartum after conception by in vitro fertilization. *Fertil. Steril* 68 492–500. 10.1016/s0015-0282(97)00230-6 9314921

[B40] Molgora S. Baldini M. P. Tamanza G. Somigliana E. Saita E. (2020). Individual and relational well-being at the start of an ART treatment: a focus on partners’ gender differences. *Front. Psychol.* 11:2027. 10.3389/fpsyg.2020.02027 33117204 PMC7549400

[B41] Monti F. Agostini F. Fagandini P. La Sala G. B. Blickstein I. (2009). Depressive symptoms during late pregnancy and early parenthood following assisted reproductive technology. *Fertility Sterility* 91 851–857. 10.1016/j.fertnstert.2008.01.021 18314111

[B42] Monti F. Agostini F. Paterlini M. Andrei F. De Pascalis L. Palomba S. (2015). Effects of assisted reproductive technology and of women’s quality of life on depressive symptoms in the early postpartum period: a prospective case-control study. *Gynecol. Endocrinol.* 31 374–378. 10.3109/09513590.2014.1000850 25625377

[B43] Moura-Ramos M. Gameiro S. Canavarro M. C. Soares I. Almeida-Santos T. (2016). Does infertility history affect the emotional adjustment of couples undergoing assisted reproduction? the mediating role of the importance of parenthood. *Br. J. Health Psychol.* 21 302–317. 10.1111/bjhp.12169 27059275 PMC5061027

[B44] Osgood C. E. Suci G. J. Tannembaum P. H. (1957). *The Measurement of Meaning.* Urbana, IL: University of Illinois Press.

[B45] Pajulo M. Savonlahti E. Sourander A. Piha J. Helenius H. (2001). Prenatal maternal representations: mothers at psychosocial risk. *Infant Ment. Health J.* 22 529–544. 10.1002/imhj.1016

[B46] Pajulo M. Savonlahti E. Sourander A. Piha J. Helenius H. (2004). Maternal representations, depression and interactive behaviour in the postnatal period: a brief report. *J. Reprod. Infant Psychol.* 22 91–98. 10.1080/0264683042000205954

[B47] Pinto T. M. Samorinha C. Tendais I. Silva S. Figueiredo B. (2017). Antenatal paternal adjustment and paternal attitudes after infertility treatment. *Hum. Reprod.* 33 109–115. 10.1093/humrep/dex349 29186413

[B48] Ranjbar F. Warmelink J. C. Gharacheh M. (2020). Prenatal attachment in pregnancy following assisted reproductive technology: a literature review. *J. Reprod. Infant Psychol.* 38 86–108. 10.1080/02646838.2019.1705261 31852259

[B49] Raphael-Leff J. (2010). “Mothers’ and fathers’ orientations: patterns of pregnancy, parenting and the bonding process,” in *Parenthood and Mental Health. A Bridge Between Infant and Adult Psychiatry*, eds Tyano S. Keren M. Herman H. Cox J. (Oxford: Wiley-Blackwell), 9–22. 10.1002/9780470660683.ch1

[B50] Registro Nazionale sulla Procreazione Medicalmente Assistita [National Assisted Reproduction Registry of Italy] (2017). Available online at: https://www.iss.it/rpma-dati-registro (accessed February 1, 2021).

[B51] Russell A. Aloa V. Feder T. Glover A. Miller H. Palmer G. (1998). Sex-based differences in parenting styles in a sample with preschool children. *Austr. J. Psychol.* 50 89–99. 10.1080/00049539808257539

[B52] Slade A. Cohen L. J. Sadler L. S. Miller M. (2009). “The psychology and psychopathology of pregnancy,” in *Handbook of Infant Mental Health*, 3rd Edn, ed. Zeanahpp C. H. (New York, NY: Guilford Press), 22–39.

[B53] Stern D. N. (1991). Maternal representations: a clinical approach and subjective phenomenological view. *Infant Ment. Health J*. 12 174–185. 10.1002/1097-0355(199123)12:3<174::aid-imhj2280120305>3.0.co;2-0

[B54] Stern D. N. (1995). *The Motherhood Constellation: A Unified View of Parent-Infant-Psychotherapy.* New York, NY: Basic Books.

[B55] Tallandini M. A. Morsan V. Macagno F. (2012). Preterm birth and assisted reproductive technology/ART: maternal emotional wellbeing and quality of mother-newborn interaction during the first three months of life. *Early Hum. Dev.* 88 397–402. 10.1016/j.earlhumdev.2011.10.003 22055247

[B56] Vitale S. G. La Rosa V. L. Rapisarda A. M. Lagana A. S. (2017). Psychology of infertility and assisted reproductive treatment: the Italian situation. *J. Psychosom. Obstet. Gynaecol.* 38 1–3. 10.1080/0167482X.2016.1244184 27750491

[B57] Vizziello G. F. Antonioli M. E. Cocci V. Invernizzi R. (1993). From pregnancy to motherhood: the structure of representative and narrative change. *Infant Ment. Health J*. 14 4–16.

[B58] Vreeswijk C. M. Rijk C. H. Maas A. J. van Bakel H. J. (2015). Fathers’ and Mothers’ representations of the infant: associations with prenatal risk factors. *Infant Ment. Health J.* 36 599–612. 10.1002/imhj.21541 26536277

[B59] Vreeswijk C. M. J. M. Maas A. J. B. M. Rijk C. H. A. M. van Bakel H. J. A. (2014). Fathers’ experiences during pregnancy: paternal prenatal attachment and representations of the fetus. *Psychol. Men Masculinity* 15 129–137. 10.1037/a0033070

[B60] Wang Y. M. Shu B. C. Fetzer S. Chang Y. J. (2014). Parenting style of women who conceived using in vitro fertilization: a meta-analysis. *J. Nurs. Res.* 22 69–80. 10.1097/JNR.0000000000000025 24821414

[B61] Wendland J. Miljkovitch I. (2003). From late pregnancy to six months’ postpartum: content and evolution of high-risk primiparous single mothers’ conscious representations. *J. Soc. Clin. Psychol.* 22 745–770. 10.1521/jscp.22.6.745.22937

[B62] Winnicott D. W. (1958). *Through Pediatrics to Psychoanalysis.* New York, NY: Basic Books.

[B63] Zeanah C. H. Benoit D. (1995). Clinical applications of a parent perception interview in infant mental health. *Child Adolesc. Psychiatr. Clin. North Am.* 4 539–554. 10.1016/S1056-4993(18)30418-8

[B64] Zeanah C. H. Benoit D. Hirshberg L. Barton M. L. Regan C. (1994). Mothers’ representations of their infants are concordant with infant attachment classifications. *Dev. Issues Psychiatry Psychol.* 1 1–14. 10.1080/14616734.2021.1876615 33480320

